# VisME: Visual Microsaccades Explorer

**DOI:** 10.16910/jemr.12.6.5

**Published:** 2019-12-12

**Authors:** Tanja Munz, Lewis Chuang, Sebastian Pannasch, Daniel Weiskopf

**Affiliations:** VISUS, University of Stuttgart, Germany; Institute of Informatics, LMU Munich, Germany; Faculty of Psychology, Technische Universität Dresden, Germany

**Keywords:** Microsaccades, visual analytics, eye movement, eye tracking, parameters fixation

## Abstract

This work presents a visual analytics approach to explore microsaccade distributions in high-frequency eye tracking data. Research studies often apply filter algorithms and parameter values for microsaccade detection. Even when the same algorithms are employed, different parameter values might be adopted across different studies. In this paper, we present a visual analytics system (VisME) to promote reproducibility in the data analysis of microsaccades. It allows users to interactively vary the parametric values for microsaccade filters and evaluate the resulting influence on microsaccade behavior across individuals and on a group level. In particular, we exploit brushing-and-linking techniques that allow the microsaccadic properties of space, time, and movement direction to be extracted, visualized, and compared across multiple views. We demonstrate in a case study the use of our visual analytics system on data sets collected from natural scene viewing and show in a qualitative usability study the usefulness of this approach for eye tracking researchers. We believe that interactive tools such as VisME will promote greater transparency in eye movement research by providing researchers with the ability to easily understand complex eye tracking data sets; such tools can also serve as teaching systems. VisME is provided as open source software.

## Introduction

Eye movements are often separated into fixations, the periods when the eyes stay rather still, and saccades, the rapid eye movements between multiple fixations. Furthermore, microsaccades (also known as “fixational saccades” (36)) are small and fast involuntary eye movements within fixations (28); they can be seen as small versions of saccades. Humans are not aware of them, but they play an important role in visual perception. Amongst other aspects, they indicate covert attention (17). Apart from microsaccades, there are other involuntary eye movements located within fixations (e.g., drifts and tremors) that are unlikely to contribute to visual information processing per se. Glissades and square wave jerks, in contrast, have a similar appearance to microsaccades, but their temporal occurrence and properties within a fixation are different. Although experienced eye movement researchers can often discriminate between these different types of eye movements manually, filter algorithms have been developed to extract them automatically and more efficiently.

For the automatic detection and labeling of microsaccades, different algorithms have been proposed (e.g., 49, 45; 5), most notably by Engbert & Kliegl (2003b)^18^. Nonetheless, the parameters for detecting and identifying microsaccades can vary immensely across different research studies. Furthermore, some studies (see Table 1 for examples) might modify basic algorithms with the introduction of additional conditional parameters (e.g., min / max amplitude, inter-saccadic interval). This lack of consistency is often tolerated in order to accommodate unavoidable variances in eye tracking data, individual behavior, and experiment designs. However, this also poses a barrier for researchers in determining whether reports of microsaccadic behavior are consistent from one study to another and the extent to which they are shaped by the chosen parameter values themselves. To facilitate the reproducibility of research results, we sought to provide a system that would support eye movement researchers in exploring and reviewing the properties of microsaccades in a given data set as well as to compare it with another. Such a system would also serve to instruct unexperienced researchers in understanding the consequences of microsaccadic filtering.

**Table 1:** to do

Paper	Method	Inter-saccadic Interval	Amplitude	Duration	λ	Binocular	Data	Other Features
Engbert & Kliegl (2003b)	New method	-	-	≥ 12 ms	6	yes	Eyelink System (SMI), 250 Hz	-
Engbert & Mergenthaler (2006)	Engbert & Kliegl (2003b)	-	≤1°	≥ 6 ms	5	yes	Eyelink II, SR Research, 500 Hz	-
Dimigen et al. (2009)	Engbert & Mergenthaler (2006)	50 ms	<1°	≥ 6 ms / 3 samples	5	no	IView-X Hi-Speed 1250, SMI GmbH, 500 Hz	-
Hsieh & Tse (2009)	Engbert & Kliegl (2003b)	80 ms	0.15°−2°	≥ 4 samples	10	-	Eyelink2, 250 Hz	(semi-)blinks and 400 ms before/600 ms after them removed
Pastukho & Braun (2010)	Engbert & Kliegl (2003b),Engbert & Kliegl (2003a)	-	-	-	-	yes	Eyelink 2000, SR Research, 1000 Hz	modified algorithm to accommodate for a higher sampling rate
Mergenthaler & Engbert (2010)	Engbert & Kliegl (2003b), Engbert & Mergenthaler (2006)	30 ms	-	≥ 6 ms	3 (free viewing); 4 (fixation task)	yes	Eyelink II, SR Research, 500 Hz	-
Bonneh et al. (2010)	Engbert & Kliegl (2003b)	-	0.08°−2°	≥ 9 ms	-	no	iViewX Hi-Speed, SMI, 240 Hz and Eyelink II, SR Research, 1000 Hz	raw data smoothed with a window of 15 ms; velocity range: 8°/s−150°/s
Benedetto et al. (2011)	Salvucci & Goldberg (2000)	-	< 1°	-	-	no	SMI X-HEAD, 200 Hz	-
Otero-Millan et al. (2012)	Engbert & Mergenthaler (2006)	20 ms	< 2°	-	6	yes	Eyelink 1000, SR Research, 500 Hz	-
Yokoyama et al. (2012)	Engbert & Kliegl (2003b)	-	-	≥ 3 samples	6	yes	Eyelink CL 1000, SR Research, 500 Hz	no trials with eye blinks or eye position more than 2° away from center
Hicheur et al. (2013)	Engbert & Mergenthaler (2006)	25 ms	< 1°	≥ 10 ms	4	yes	Eyelink 1000, SR Research, 1000 Hz	microsaccades within 50 ms after a saccade were not considered as microsaccades
Pastukhov et al. (2013)	Engbert & Kliegl (2003b)	-	< 60′	-	-	yes	Eyelink 2000, SR Research, 1000 Hz	square wave jerks
Di Stasi et al. (2013)	Engbert & Kliegl (2003b)	20 ms	< 1°	≥ 6 ms	6	yes	Eyelink 1000, SR Research, 500 Hz	(semi-)blinks and 200 ms before/after them removed
McCamy, Najafian Jazi et al. (2013)	Engbert & Kliegl (2003b)	20 ms	< 2°	≥ 6 ms	4	yes	Eyelink 1000, SR Research, 500 Hz	(semi-)blinks and 200 ms before/after them removed
Costela et al. (2013)	Engbert & Kliegl (2003b)	20 ms	< 1°	≥ 6 ms	4	yes	Eyelink 1000, SR Research	(semi-)blinks and 200 ms before/after them removed
McCamy, Collins et al. (2013)	Engbert & Kliegl (2003b)	20 ms	≤ 2°	≥ 6 ms	6	yes	Eyelink II, SR Research, 500 Hz	(semi-)blinks and 200 ms before/after them removed
McCamy et al. (2014)	Engbert & Kliegl (2003b), Engbert & Mergenthaler (2006)	20 ms	< 1°	≥ 6 ms	6	yes	Eyelink II, SR Research, 500 Hz	(semi-)blinks and 200 ms before/after them removed
Privitera et al. (2014)	Engbert & Kliegl (2003b)	-	< 1.2°	-	6 and 3	no	Eyelink 1000, SR Research, 1000 Hz	first peak velocities were determined then their extent
Yuval-Greenberg et al. (2014)	Engbert & Mergenthaler (2006)	-	< 1°	-	6	-	Eyelink 1000, SR Research, 1000/500 Hz	-
Fried et al. (2014)	Bonneh et al. (2010)	yes	> 0.1° ∧ ࣘ≤ 2°	≥ 6 ms	-	-	Eyelink 1000, SR Research, 500 Hz	(semi-)blinks and 20 ms before/after them removed; min velocity: 10°/s; peak velocity: > 18°/s
Poletti & Rucci (2016)	-	15 ms	3′−30′	-	-	yes	-	-
Krejtz et al. (2018)	Engbert & Kliegl (2003)	-	-	≥ 6 ms	6	no	Eyelink 1000, SR Research, 500 Hz	microsaccades detected within fixations; average position of right and left gaze points are used
Summary	-	0−80 ms		6−16 ms	3 - 10	yes/no	200 - 1000 Hz	min velocity: 8°/s−18°/s; max velocity: 150°/s; vel. window: 5−15 ms; ignore time after saccade: 0−50 ms; (semi-)blinks and 200 ms before/after them; ...

Recent years have witnessed an increase in the adoption of advanced visualization techniques by eye tracking researchers (see 7, for a survey). However, no visualization techniques have been designed specifically for the analysis of microsaccades. Conventional visualization techniques tend to focus on low-frequency data (i.e., dwells, fixations, and saccades); for instance, scanpaths that visualize sequences of fixations and saccades (e.g., 28) or attention maps (e.g., 60). Visualizations can communicate derived statistics, which are often more informative than simply plotting the raw data, such as summary statistics of microsaccade direction and amplitude. Polar plots and rose plots are common methods to indicate the distribution of eye movement directions (e.g., 18; 34; 59; 37, 54, 23). Scatterplots are useful in depicting the main sequence relationship between peak velocities and microsaccade amplitudes (e.g., 18) and histograms illustrate the data set’s distribution of microsaccade peak velocities, magnitudes, or durations (e.g., 51). Otero-Millan et al. (2008) and McCamy et al. (2014), for example, use figures where raw data samples are plotted on top of the stimulus, highlighting microsaccades. In order to show temporal positions of microsaccades in relation to the eye movement, timelines are employed by McCamy et al. (2015) and Otero-Millan et al. (2014).

Eye tracking data are oftentimes large data sets of time series prior to feature extraction. With visualization alone, it might be difficult to handle all of the data and to fully understand it. Visual analytics can be an invaluable tool in allowing researchers to understand and compare complex data sets (11). According to Thomas and Cook (2005), “visual analytics is the science of analytical reasoning facilitated by interactive visual interfaces.” Following their definition, visual analytics (61; 31; 12) can be a useful choice in the development of an eye tracking exploration system; different techniques are used by people to gain insights. In particular, interaction is an important addition to visualizations for complex data analysis and to support the analytical process. Interaction and filtering allow the user to extract important information and to further explore it. For example, interaction techniques such as brushing-and-linking (62) can allow researchers to connect corresponding data points across different data visualizations for the same data set. Furthermore, interactive filtering can allow researchers to vary the type and amount of data used for visualization in real time. For eye tracking data in general, different visual analytics methods have been investigated by Andrienko et al. (2012). Kurzhals and Weiskopf (2013) introduce a visual analytics method for dynamic stimuli. To the best of our knowledge, no visual analytics system has been developed for the visual exploration of microsaccade behavior that takes eye tracking data as input and allows for the real time adaptation of filter algorithms.

Studies on microsaccadic behavior are often highly controlled psychophysical experiments (e.g., 18; 34; 25), as opposed to studies that involve natural viewing tasks (c.f., 64; 24). As far as we know, no specific visualization software system exists to support the exploration of microsaccade distributions of eye movement data sets for the natural viewing of complex scenes. Our approach allows the analysis of both types of experiments. Generally, VisME is agnostic to experiment design and stimulus. This means that it can also support the comparison of data sets across different studies.

In this paper, we present an interactive visual analytics system for high-frequency eye tracking data, with a focus on microsaccade exploration. We provide different visualization and interaction techniques to visualize conventional properties of microsaccade behavior (i.e., amplitude, direction, peak velocity, duration, and temporal and spatial distribution). In an analytical process, a combination of these techniques can be used for the exploration of eye tracking data sets. One key property of this system is that data visualizations can be achieved on different levels of analysis (i.e., fixation, trial, and test condition level) for individuals as well as groups of participants. The analysis is supported by brushing-and-linking and different visualizations that are shown simultaneously in multiple views. We have added the following visualization types to VisME to understand the relationship between space, time, and movement: stimulus view, timeline view, polar / rose plots, histograms, and scatterplots. Thereby, this is the first technical solution that allows users to interact and dynamically explore eye tracking data for the statistics of microsaccadic behavior. Given its popularity, we adopted the microsaccade detection algorithm by Engbert and Kliegl (2003b) as a starting point and included additional features to allow for interactive parameter control. Using our system, researchers are encouraged to vary these parameter values continuously in order to graphically compare their impact on the data set. In a what-if analysis, researchers are able to explore data sets and the influence of parameters. We present in a case study how this system can be employed on different eye movement data sets for natural scene viewing and demonstrate how interactive filtering allows researchers to inspect influences on the number and distribution of microsaccades across different levels (i.e., for fixations, participants, trials, and test conditions). In a usability study, we collected feedback from eye tracking researchers. They agreed that our system is useful for teaching purposes and eye tracking research alike. In particular, we report how visual analytics tools like VisME can promote data transparency, which is consistent with the current aims of open science (48).

This work contributes by simplifying the exploration of microsaccadic data sets through interactive visual analytics. First, our system can be used to better understand how changes in the parameter values of microsaccade filters can influence the spatial and temporal distributions of microsaccades. Next, it is convenient for general visual exploration of microsaccades using interaction techniques like filtering to analyze microsaccades on different levels. In line with the increasing availability of eye movement data sets, our visual analytics system will help researchers and their reviewers critically discuss and (re-)analyze data. We provide the source code of our system (Munz, 2019) on GitHub: https://github.com/MunzT/VisME.

In the next sections, we first provide some domain background and requirements for the system we propose. Then, we describe features of VisME, the visual analytics system we developed for analyzing microsaccades. In a subsequent case study, we demonstrate for two externally collected eye tracking data sets how our system can be used and present the qualitative feedback we collected from eye tracking experts in a usability study. A short conclusion and ideas for future work finalize our paper.

## Methods

In this section, we provide details on the definition of microsaccades in the eye tracking domain and the requirements for the system we implemented. Afterward, we give an overview of our system’s features.

We used a formative process for the development of our design, following the nested model by Munzner (2009). We focused on the outer parts of the model: domain problem characterization, data / operation abstraction design, and especially the encoding / interaction design. The process was performed in close cooperation with an eye tracking expert. In multiple sessions over a period of 16 months, our system was repeatedly refined.

### Background to Microsaccade Detection

The parameter settings of filter algorithms are crucial for the detection of eye movements (2; 8; 30). Poletti and Rucci (2016) addressed the challenges of defining microsaccades. Table 1 shows how the chosen parameter values and algorithmic features have differed across recent studies (i.e., 2009–2018) on microsaccades (a similar table for the period of 2004–2009 is provided by Martinez-Conde et al., 2009). In particular, Table 1 summarizes how the critical parameter values of the algorithm by Engbert and Kliegl (2003b) (e.g., λ, which is used to calculate velocity thresholds) vary across published studies. It is noteworthy that even the original values chosen by Engbert and Kliegl (2003b) and Engbert et al. (2006) differed, in order to accommodate for irrelevant variances across their different data sets. For example, Malinov et al. (2000) detected only two eye movements as microsaccades out of 3375 saccades using a value of 0.2° as maximum amplitude. In Table 1, it is visible that for all experiments larger amplitudes were used.

Eye movements that are similar to microsaccades (i.e., glissades and square wave jerks) can be detected with the same algorithms (28) and are treated according to the researchers’ discretion. Square wave jerks are involuntary eye movements that move the eye first away from the visual target and then back onto it; they consist of two small saccades in opposite directions and some latency in-between (28). Glissades are post-saccadic eye movements before the eye comes to a halt; depending on the fixation filter, they are assigned to either fixations or saccades (28). In the literature, the term glissade might occasionally be applied with different definitions (e.g., 4; 63; 14). In the following, we regard as glissades all types of high-velocity over- and undershoots directly succeeding saccades (28). Abadi and Gowen (2004) do not differentiate between microsaccades and glissades; Hafed and Clark (2002) handle square wave jerks as a pair of microsaccades in opposite directions. 

Given that the extraction of microsaccades can differ across different studies, it is unclear if findings can be expected to reasonably generalize from one study to another. It is often unclear why specific parameter values have been chosen, or if other values might have produced different results. Thus, VisME was developed to support researchers in inspecting the influence of parameter variations.

### System Requirements

We identified the following requirements for our application to explore microsaccades, serving as a basis for the visual encoding and interaction design:

Ability to:

explore microsaccades in the context of the entire eye tracking data in time and space.

explore the relationship between space and time for microsaccades.

explore microsaccadic properties.

explore individuals and groups of participants.

explore the location of microsaccades within fixations.

change parameters for microsaccade detection.

study statistical values when changing parameters.

differentiate between microsaccades and similar eye movements.

explore the influence of fixation filters on microsaccades.

explore the relationship between microsaccades and test conditions.

integrate VisME into existing analysis pipelines.

We believe that interactive visualization along with data filtering is a most appropriate approach to support scientists exploring these aspects in a visual analytics system.

### Visual Analytics System

**Figure 1.**
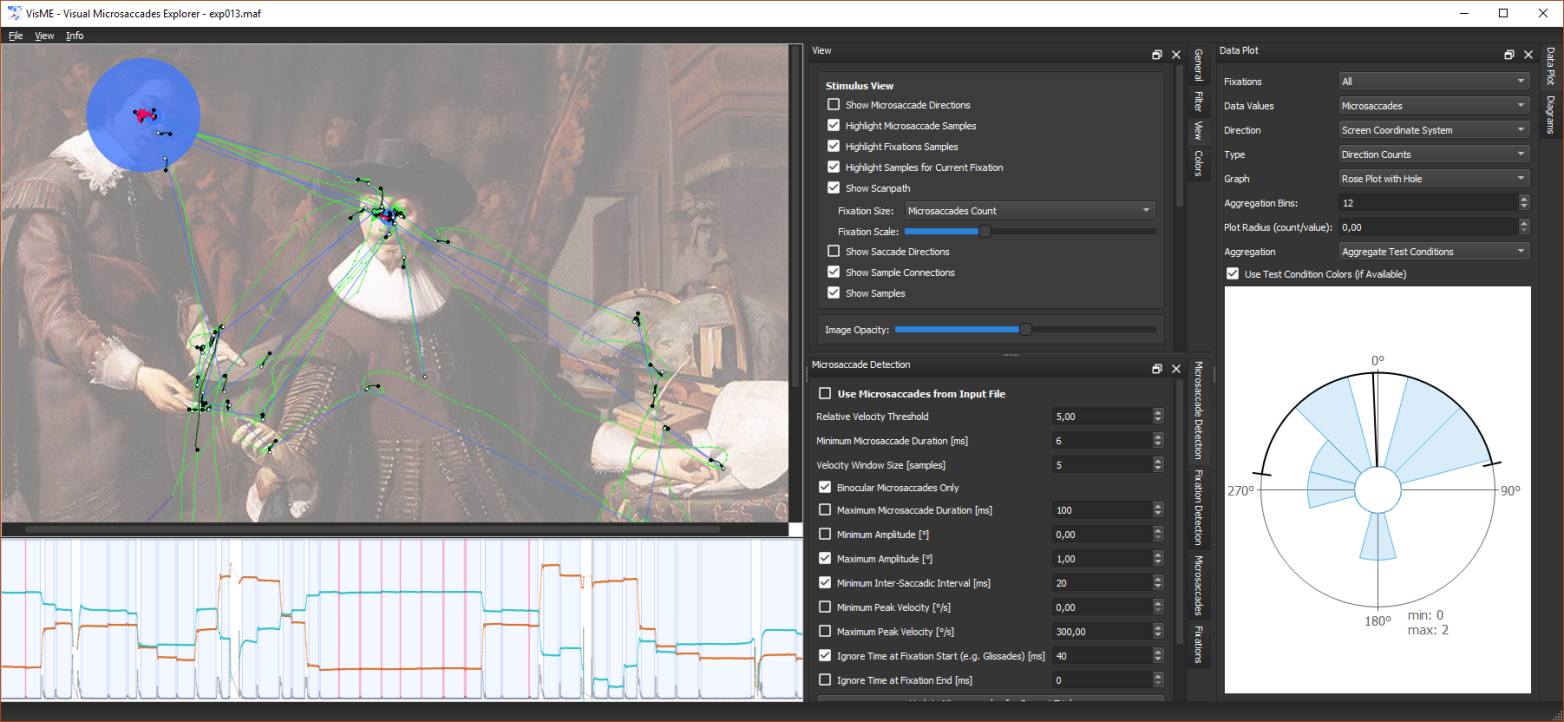
Screenshot of the user interface showing visualizations of high-frequency eye tracking data in three linked views: (top left) gaze positions with highlighted fixation and microsaccade samples on top of the stimulus, (bottom left) temporal dependency of the eye movements and microsaccades, (right) rose plot of microsaccade directions.

In this section, we detail the implemented filters for microsaccades and fixations, and the different visualization and interaction techniques employed to explore high-frequency eye tracking data. For the analysis, fixations have to be determined first. Afterward, microsaccades can be detected within their time ranges and different views can be used for visualizations and interaction. The general user interface design can be seen in Figure 1.

### Eye Movement Filters

Eye tracking data can be grouped into different eye movement classes. Often, these movements can be pre-calculated by the eye tracking system itself. In VisME, it is possible to explore eye movements (fixations and microsaccades) that were determined in pre-processing or with the system itself. In the following paragraphs, we describe the filters we use in our application to interactively label microsaccades and fixations.

**Microsaccade Filter** – In contrast to detecting fixations, eye trackers do usually not provide filters for microsaccades. For interactive microsaccade detection, we chose the velocity-threshold algorithm by Engbert and Kliegl (2003b) as an example; other algorithms could have been used as well. A free parameter λ is used for a velocity threshold in 2D space and a minimum microsaccade duration is also fixed.

Available parameters for microsaccade detection in our system are:

λ for the velocity threshold

minimum and maximum duration

minimum and maximum amplitude

minimum and maximum peak velocity

velocity window size

time being ignored at beginning / end of fixations (e.g., to ignore glissades)

time being ignored after a microsaccade, i.e. minimum inter-saccadic interval (to ignore overshoots)

time being ignored before / after missing data

monocular or binocular microsaccades

We decided upon these parameters as they were prominently mentioned in previous work (see Table 1). Initially, we set the parameters in our system according to the values mentioned by Engbert and Mergenthaler (2006) (λ = 5, minimum duration = 6 ms, velocity window size = 5 samples, binocular microsaccade detection, maximum amplitude = 1°) and set minimum inter-saccadic interval to 20 ms and ignored the first 20 ms of fixations.

**Fixation / Saccade Filter** – Our method uses the same algorithm that we implemented for detecting microsaccades but with different default parameter values (λ = 8, minimum saccade duration: 3 ms, velocity window size: 9 samples, minimum saccade amplitude: 1°, minimum inter-saccadic interval: 50 ms). This method is a saccade filter and fixations are defined as the intervals between two saccades. Mergenthaler and Engbert (2010), Otero-Millan et al. (2008), Sinn and Engbert (2011), and Laubrock et al. (2010) also used this algorithm to detect saccades. When applying different fixation filters or parameter values, it is possible that some fixations are not detected at all or multiple fixations are detected as only one fixation. While one algorithm detects two fixations connected by a saccade, another might detect just one fixation that contains a microsaccade. This, of course, influences the relationship between microsaccades and fixations. Also, if more or fewer data samples are included within a fixation, the center of a fixation shifts.

### Visualizations

To explore the distribution of microsaccades, our application provides multiple linked views that can be explored interactively: A stimulus view, which shows the stimulus, the raw data, and a scanpath visualization; a timeline view for visualizing the temporal distribution of microsaccades in relation to the eye movement; data plots used for visualizing descriptive statistics and details on microsaccade distribution; and histograms and scatterplots for further microsaccadic properties.

**Figure 2.**
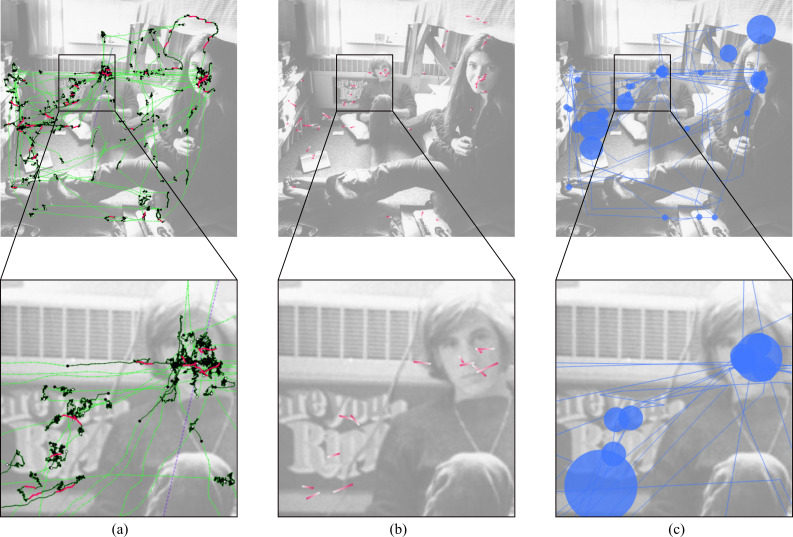
Different aspects of the eye tracking data can be visualized in the stimulus view. (a) Connected raw samples (green), connected fixation samples (black), connected microsaccade samples (pink), and missing data range (purple). (b) Directional microsaccade lines: pink for start sample, white for end sample. (c) Scanpath (blue) with fixations represented by circles (their size is in relation to the number of microsaccades within them) and saccades by lines.

**Stimulus view** – The stimulus view provides an overview of the eye tracking data. In this view, all raw eye tracking samples, fixation samples, microsaccade samples, and missing data ranges can be shown or highlighted (Figure 1 (top left) and Figure 2 (a)). A plot with directional microsaccade distributions can be displayed with lines that connect the start and end samples of microsaccades colored with a gradient to encode the directions and locations of the microsaccades on the stimulus (Figure 2 (b)). A common scanpath visualization for fixations and saccades is also available (Figure 2 (c)). The size of the fixations can be either in relation to the duration of fixations, the number of microsaccades within fixations, or in such a way that each circle has the same size. Using filters, it can be decided which information shall be visible in this view.

**Figure 3.**
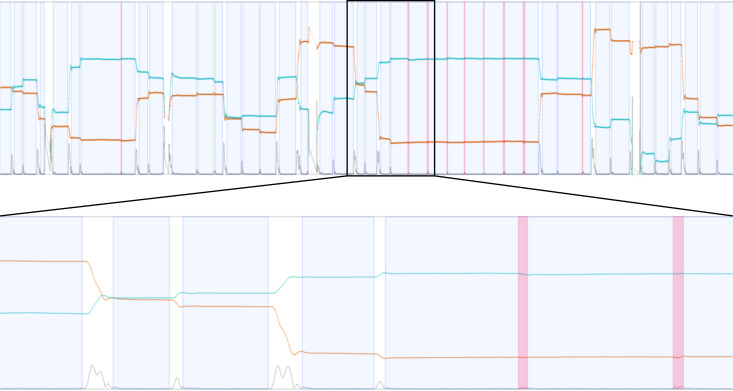
Timeline view: timeline (top) and zoomed timeline (bottom) to explore more details. Timelines show the eye positions in x (orange) and y (turquoise) direction, the velocity (gray) determined by these positions, missing data ranges (purple) in x and y direction, fixation areas (blue), and microsaccade locations (pink).

**Timeline** – The timeline gives a better understanding of the relationship between time, the eye positions, and eye events. It is visible where fixations are located and microsaccades appear. As some experiments depend on specific temporal events, event positions can be highlighted as well. This view is zoomable to see more details of the surroundings of microsaccades and fixations, see Figure 1 (bottom left) and Figure 3.

**Figure 4.**
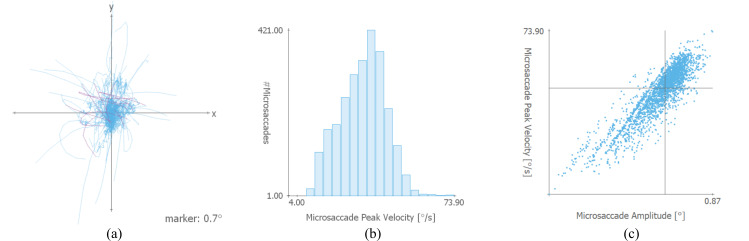
(a) Position of microsaccades relative to fixations of a trial in the context of the fixations that are plotted on top of each other (microsaccades are highlighted in pink). The marker value indicates the extent at the marked position on the x and y axis; values are measured in visual degree. (b) Histogram for microsaccade peak velocity and (c) the relationship between amplitude and peak velocity as scatterplot using a logarithmic scale for the painting data set.

**Data Plots** – In a separate section of our system, descriptive statistical graphics (e.g., rose plots) and further fixation-related visualizations are used to show more details on the data set in relation to microsaccades and the chosen parameters. Rose plots (see Figure 1 on the right side) or polar plots are used to show the direction of microsaccades; the data is aggregated to a specified number of bins (if not mentioned otherwise, 12 bins are used in all graphics). If multiple test conditions are specified, data of each test condition type can be visualized with another color value. Additionally, the mean direction and standard deviation are shown in black. To see the microsaccade distributions, especially the number and locations within fixations, the plot type can be changed; see Figure 4 (a) for an example. In this image, all fixations of a trial are plotted on top of each other; microsaccades are highlighted in pink. A similar plot that can be created with VisME shows only microsaccade directions in relation to the fixation center. The same start and end points of microsaccades are used as shown in the image, and a color gradient is applied to indicate their direction similar to the direction plots in the stimulus view.

While all the data visualized in this area can be shown with microsaccade directions in screen coordinates, it is also possible to transform the data in such a way that directions of microsaccades are rotated toward the next fixation. For these plots, the direction to the next fixation points to the top (e.g., 0° in rose plots). This transformation is useful for a better understanding of microsaccades and the whole eye movement. To determine the relationship toward the next fixation, a direction vector between two fixation centers is used. For the rose plots, a microsaccade within the first fixation is translated in such a way that its start point is located at the center of the corresponding fixation. The angle between the vector of a microsaccade (from start to end sample) and the direction vector determine the angle used for the direction. For the other data plots, the direction vector determines how all data of a fixation has to be rotated.

**Histograms and Scatterplots** – A further area of the system provides the possibility to explore the temporal locations, durations, amplitudes, and peak velocities of microsaccades in histograms and scatterplots (Figure 4 (b) and (c)). Especially scatterplots that show the relationship between amplitude and peak velocity are commonly used for both saccades and microsaccades to explore the main sequence. Additionally, histograms show the temporal distribution of microsaccades within fixations.

### Interaction Techniques

Our visual analytics system provides many interaction methods to explore the raw data on different levels, i.e., for fixations, participants, trials, and test conditions. Interaction supports the analysis process for microsaccades for which we will show some examples in our case study. With brushing-and-linking, a combined perspective of different aspects of the data shown in different views can be obtained. The parameters for detecting microsaccades can be modified, the visible data can be filtered on different levels, and standard navigation techniques such as zooming and panning are available. It is possible to select which eye data (left / right / averaged) should be used for analysis and a rubber band is available to select a time range in the timeline. The filters allow exploration of the data on different levels: for participants, trials, and test conditions. Furthermore, the amount of data visualized in the different views can be adapted by hiding different elements. As all views are linked, it is possible to select a fixation (if a single trial is being explored) in either time, space, or a list of all fixations to highlight this fixation; it will be highlighted in the other views as well, the corresponding data plots will be shown, and some details on the fixation will be displayed. Additionally, it is possible to walk through the scanpath for every fixation. As it is also of interest to see relationships of sequential fixations, it can be specified how many neighboring fixations shall be visualized and the data plots will include these fixations as well. Moreover, some statistical values about the selected trials are updated when parameters change. These include information about fixations (e.g., count, duration, or percentage containing microsaccades) and microsaccades (e.g., count, duration, amplitude, peak velocity, count per second / fixation, or inter-saccadic interval). 

### Data Import and Export

Our application uses its own format for input data to be independent of any eye tracker. High-frequency eye tracking data is required as input, with a minimum frequency of 200 Hz in order to detect microsaccades (Holmqvist et al., 2011). For each participant, a separate file is required that can contain multiple trials specifying raw samples, fixations, microsaccades, and event locations; a second file type can contain information about test conditions (e.g., tasks or other trial-specific circumstances). Files with the current (possibly calculated) eye movement data (raw data, fixations, microsaccades, and events) can be exported for further analysis with other statistical software such as R, Python, or MATLAB and for later import into VisME. Additionally, aggregated statistics for different properties of fixations and microsaccades containing data for each participant and test condition can be exported for analysis in other applications. More details about the file formats used in VisME are available by Munz (2019) and on GitHub (https://github.com/MunzT/VisME).

Our system can be integrated in full study and analysis pipelines. Researchers have to convert their eye tracking data into our expected input format. It is possible to import detected microsaccades determined in other steps of their analysis to verify their properties or to calculate a new set of microsaccades with VisME by visually inspecting the result. Our system can then be used to explore the data and to export the raw data along with detected microsaccades as well as aggregated data for further processing.

### Implementation Details

Our application is platform-independent and was tested on Windows 10 and Linux. It is implemented in C++ with Qt 5.9 using the Graphics View Framework for interactive visualizations. We reimplemented the R code available by Engbert et al. (2015) to detect microsaccades and added some additional conditions as described previously.

In pre-processing steps for the case study and user study, movements recorded by an EyeLink eye tracker were first converted from .edf files to .asc files using the converter available by SR Research: the EyeLink EDF2ASC Converter. Then, we used a script written in Python to create an input file in the format expected by our application, containing eye tracking positions and fixations.

The source code of our implementation, a detailed description of the input and output formats and the Python script are provided by Munz (2019) and on GitHub together with a more detailed manual for the system, so that the system can be readily used and the data file converter can be easily adapted for other raw data. Currently, only the microsaccade filter as described before is implemented, but the system is designed that other methods can be implemented and added with little effort. In order to explore microsaccades detected with other algorithms, the import of the data into our system is possible.

## Results

To demonstrate how our application can be used, we use externally collected high-frequency eye tracking data of two independent experiments for our case study and in a usability study. 

### Eye Movement Data Set

In both experiments that were conducted to collect the two data sets, participants were asked to look at images with some given task for the exploration. Eye movements were recorded with remote eye trackers (Eyelink 1000, SR Research) using a chin rest to stabilize heads of participants. Fixations were determined by the eye tracking software of the systems. While for the first data set monocular data is available, the second data set allows binocular analysis as well.

As a first data set (photo data set), we use data collected from experiment 3 of Greene et al. (2012). It contains eye tracking data on 16 participants who viewed 20 grayscale natural images for 60 seconds whilst performing one of four tasks. The tasks were: memorize the picture (memory), identify the decade in which the picture was taken (decade), assess how well the people in the picture know each other (people), and determine the wealth of the people in the picture (wealth). The data was recorded at 1000 Hz and only the right eye was tracked. The data set was originally created to verify Yarbus’s assumption that the eye movement is highly influenced by an observer’s task. We chose this data set because it is high-frequency data that allows extraction of microsaccades and participants performed different high-level cognitive tasks that were likely to have engaged covert attention even if the study was not designed to investigate this aspect of gaze behavior. Many researchers believe that covert attention can influence the frequency of microsaccades and their directions (e.g., 18; 19; 25; 26; 52; 34).

The second data set (painting data set) was recorded to explore the occurrence of microsaccades in free-viewing conditions. It is available at 500 Hz for both eyes. Gaze samples for averaged eye movements were determined as the mean value of right and left eye positions and fixations as the maximum fixation areas of both eyes. 20 participants looked at 60 randomized colored paintings showing multiple persons for about 15 seconds per image. The participants’ task was to pay attention to the presented people, their mood, and relationship to each other (acquaintance) and they had to answer questions afterward.

### Case Study

In our case study, we demonstrate how it is possible to visually explore the directions and distributions of microsaccades with VisME. Additional examples and details are provided in the supplemental material.

We use the initial parameter values mentioned before for our exploration.

**Figure 5.**
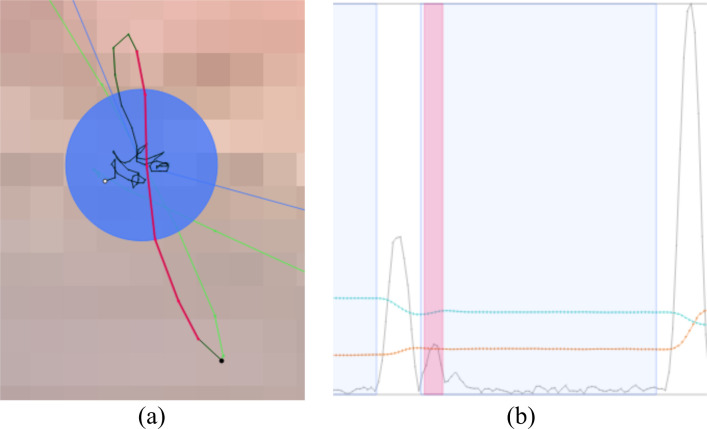
Eye movement detected as microsaccade (pink); it can be identified as glissade on the stimulus and in the timeline: (a) Connected samples that belong to the microsaccade are located at the beginning of the fixation (black). (b) Zoomed view of the timeline: the detected microsaccade is located at the beginning of a fixation (blue) right after a saccade. (x values: orange, y values: turquoise, velocity: gray). Example used from the painting data set.

**Figure 6.**
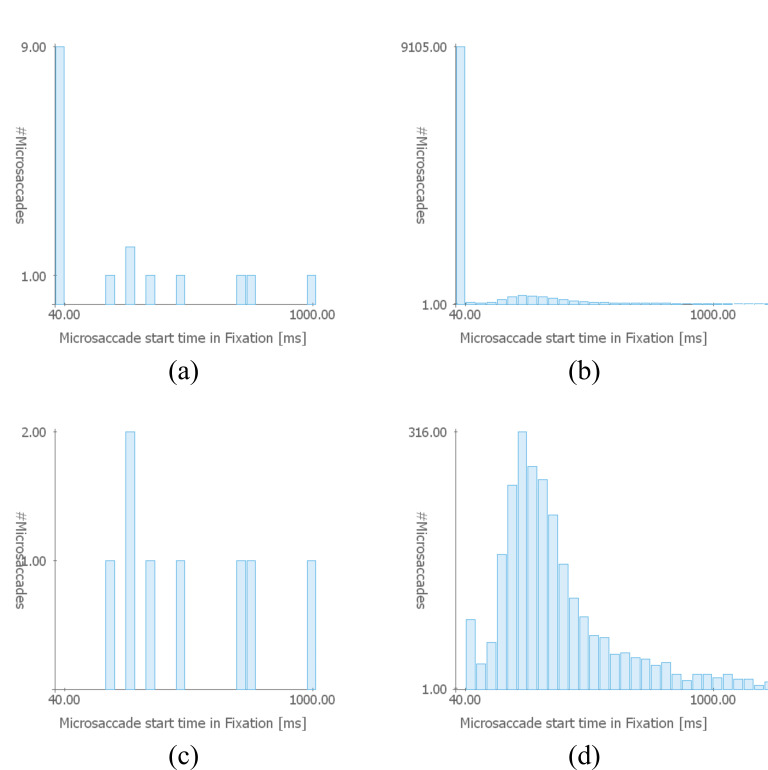
Temporal positions of detected microsaccades as a histogram for (a) one trial and (b) all trials of all participants in the painting data set (bin size: 40 ms). A high peak in the first bin indicates that many glissades (overshoots right after saccades, here in the subsequent 40 ms) were detected as microsaccades. Figures (c) and (d) show the same data when all microsaccades within the first 40 ms of a fixation are ignored from the analysis.

**Detecting Glissades** – Glissades are eye movements that immediately succeed saccades. In order to demonstrate the confusability of microsaccades and glissades at the start of fixation periods, we changed the potential glissade duration to 0 ms; this is an adjustable parameter in VisME. Subsequently, we inspected the stimulus view and timeline with highlighted microsaccades of one trial. We noticed that many detected microsaccades were both spatially and temporally located at the beginning of fixations, right after saccades. Velocity peaks also indicated that they might be microsaccades. However, as they are located right after saccades, they are more likely to be glissades (see Figure 5). Additionally, we had a look at the histogram for the temporal location of microsaccades within fixations (Figure 6 (a) and (b)): it is visible that for both the current trial and the whole data set there is a high peak for potential microsaccades within the first 40 ms, which might be glissades instead. This suggests that this phenomenon is present in the whole data set. Thus, we modified the parameter for the potential glissade duration from 0 ms to 40 ms for the painting data set for the remaining part of the analysis. Note that this parameter would be arbitrarily determined by most researchers with no opportunity for re-analysis by others, in the absence of a tool like VisME. In Figure 6 (c) and (d), the early fixation period was excluded from the analysis, which resulted in a more suitable distribution of detected microsaccades. There is still a peak in the second bin (Figure 6 (d)) but visibly diminished relative to the previous first bin. This could explain why Hicheur et al. (2013) did not consider microsaccades within even 50 ms after a saccade. We noticed this behavior in both data sets; for the photo data set, we chose a threshold of 20 ms to obtain a similar distribution. Depending on the chosen fixation filter and possible other reasons we are not aware of, this value might have to be chosen differently for other data sets. Our system can be used to find a suitable minimum time range to remove glissades without removing actual microsaccades from further analysis.

**Figure 7.**
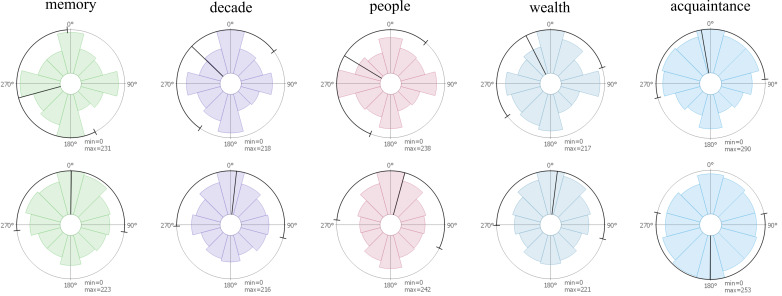
Rose plots for microsaccade distributions for the 4 different tasks of the photo data set (memory, decade, people, and wealth) and the painting data set (acquaintance). First row: directions of microsaccades in screen coordinates as visible on the stimulus. Second row: microsaccade directions in local coordinates: they are rotated in such a way that 0° means that a microsaccade is in the direction to the next fixation. Min and max specify the values in the middle of the plot and on the circle, respectively; values are measured in microsaccade count.

**Microsaccade Directions for Test Conditions** – To visualize the microsaccade directions across different tasks, rose plots were employed to illustrate, for all available trials, the distribution of microsaccades in every direction (Figure 7). This reveals potential asymmetry in the circular distribution of microsaccadic movements. We use both data sets and differentiate trials for the photo data set by different tasks and for the second one we visualize all data together. In the first row, the photo data set reveals a tendency for microsaccades to be oriented horizontally and vertically within the image. For the painting data set, microsaccadic movements were biased toward the top, which could be indicative of a recording bias. The rose plots in the second row illustrate the directions of microsaccades relative to the next fixation (toward the next fixation means to the top of the graph). The photo data set revealed a tendency of microsaccades toward the next fixation (especially for the task people), indicating that microsaccades predict the next fixation. Additionally, there is also a strong tendency toward the opposite direction. For the painting data set, a similar vertical bias was visible that was less pronounced and favored the opposite direction to the next fixation. In these plots, no correlation between the two tasks related to people (people and acquaintance) are visible, but a similarity of data recorded in the same experiment can be seen. A possible reason why these different results are obtained might be due to data quality and existing noise when using monocular data.

**Figure 8.**
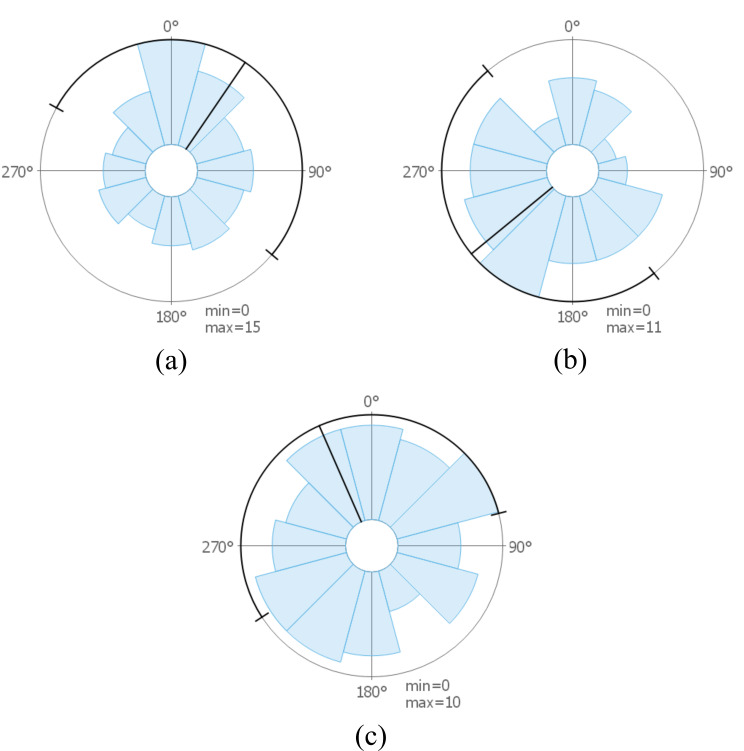
Rose plots showing microsaccade directions rotated toward the next fixation for a selection of participants from the painting data set. Participants whose microsaccades have a direction that (a) tend to be toward the next fixation, (b) toward the opposite direction, and (c) that are equally distributed toward each direction.

**Microsaccade Directions for Participants** – Our next step was to examine the data on an individual level for different participants. For selected participants, images are visible in Figure 8. It is possible to see how data can vary between different participants: the images show results for participants that might indicate that microsaccades move toward the next fixation, the opposite direction, or in arbitrary directions. Such visualizations could support researchers in identifying different microsaccade patterns available for certain participants and compare if patterns are similar to the aggregated directional distribution of all participants (see Figure 7 (acquaintance, bottom)) or rather outliers. 

**Figure 9.**
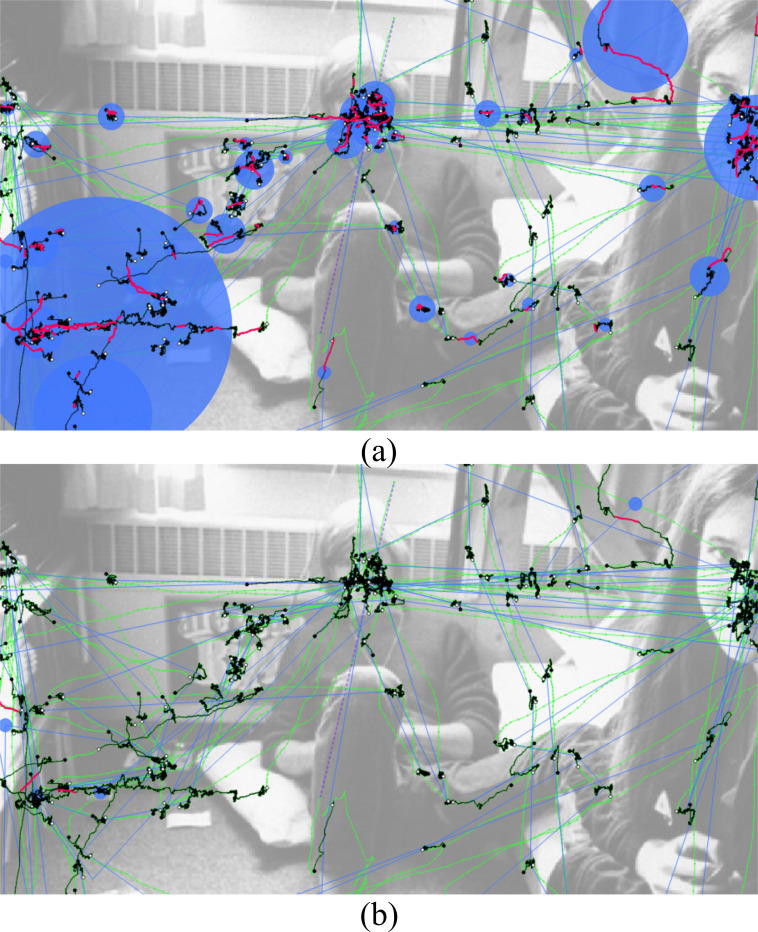
(a) Result of the microsaccade filter when changing all parameter values in such a way that more microsaccades can be detected using values in the range as given in Table 1 and (b) when using parameter values to limit the detection. A trial from the *photo* data set is shown.

**Microsaccade Detection for Changed Parameters** – In order to see the strong influence of parameters on the detection of microsaccades we used two different settings of parameter values taken from the value ranges visible in Table 1: the first settings contain values that result in many microsaccades (minimum inter-saccadic interval: 80 ms, maximum amplitude: 1°, minimum duration: 12 ms, λ = 8, velocity window: 5 samples, ignore time at beginning of fixations: 50 ms; parameters not mentioned were deactivated) and the other ones in few (maximum amplitude: 2°, minimum duration: 6 ms, λ = 3, velocity window: 5 samples). The use of the first parameter settings missed many actual microsaccades and the second one detected many false microsaccades. The difference in the number of detected microsaccades varied for the trial visible in Figure 9 from only 4 to 184 (if glissades were excluded (here, the first 20 ms of a fixation), still 138). It is apparent that there is a large range of possible sets of microsaccades that can be detected with values chosen between these two different settings. The visual analytics system can help in exploring the data to choose appropriate values for a given data set. A visual inspection in the stimulus view is necessary to confirm if the parameter changes improve the detection of microsaccades or result in false positives or missed microsaccades. 

To further explore the influence of parameter choices, we provide as supplemental material an overview of polar plots, histograms, and main sequences for both data sets when changing individual parameter values to the maximum or minimum values given in Table 1. We created aggregated statistical distributions for all participants and trials to show how they change when adapting parameter values. In the following, we compare the results of our default settings to some adaptions. We can see a strong influence for different velocity threshold (λ) values. Mergenthaler and Engbert (2010) use λ = 3 for free-viewing experiments. Using the same value for our data sets shows an increase in the number of detected microsaccades by a factor of three. The main direction changes toward the bottom and there are especially more detected microsaccades with smaller velocity, amplitude, and duration. Some of them are also located at a later position during a long fixation. When using λ = 10 (29) instead, fewer microsaccades are detected, which are mostly moving to the left and right side. The number of microsaccades is reduced for small peak velocities, high duration, and small amplitudes. When the threshold for the minimum duration is increased, fewer microsaccades with smaller duration and smaller amplitude are detected. Changing the size of the velocity window (9) shows only for the painting data set a noticeable different pattern. In the main sequence, we see microsaccades with very large velocity values; this might be an indication for noise. The other plots also show slightly different patterns (e.g., the duration histogram seems to be shifted). As the painting data set was recorded for both eyes, we can compare the influence of using monocular or binocular detection. Using the monocular detection, more microsaccades are detected. There is an increase of microsaccades with higher peak velocity, smaller duration, and smaller amplitude. We can also see that many microsaccades have a direction toward the bottom of the screen.

**Figure 10.**
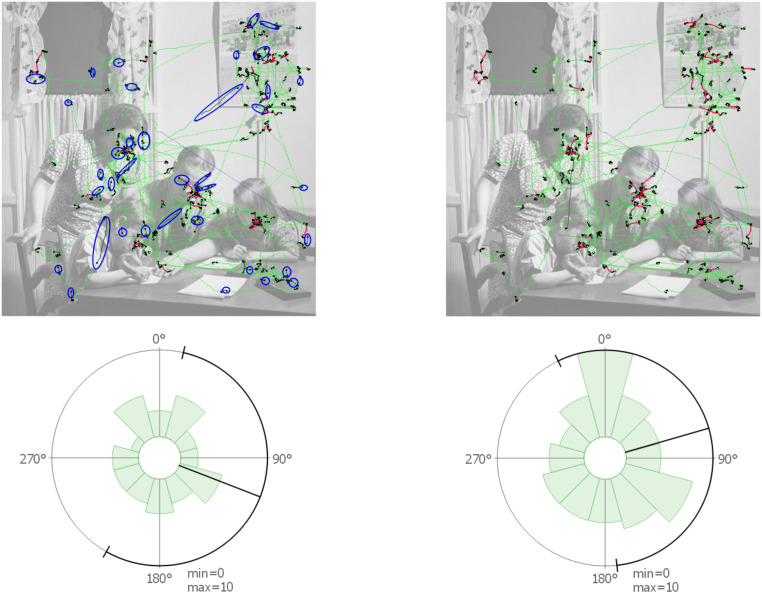
Usage of another fixation filter influences the sample ranges used for microsaccade detection and thus the rose plot visualizations. The left images use the fixations from the eye tracker, the right ones the fixations calculated by the application. Microsaccade detection was performed in both cases using the microsaccades filter with default parameter values. In the first row, some differences in the detected fixations are highlighted in blue. In the second row, it is visible that the microsaccade distribution for the trial changes as well.

**Influence of Fixation Filters** – As the fixations themselves also influence microsaccades, we used the fixation filter of our system (see section Eye Movement Filters for details) to detect different fixations than the ones already determined by the software of the eye tracker. In Figure 10, the raw data with highlighted fixation and microsaccade samples is visible for an example scene. The left images were created for the fixations provided by the eye tracker, the right ones for the built-in fixation filter. It is visible that the fixations change most notably in size and hence the microsaccade amount and directional pattern change as well. 

In our exploration process, we observed that microsaccade distributions depended on different factors. Fixation labeling defined the data that was used for the extraction of microsaccades and defined the spatial relationship between microsaccades and the next fixation. Microsaccade parameters are important for the calculation of microsaccades within these fixations. They influence, for example, if confusable eye movements (e.g., glissades) will be regarded as microsaccades, which has a strong influence on the overall statistics. By looking at individual trials, it is possible to determine if small microsaccades were missed or if fixation samples might have been mislabeled as microsaccades. Overall it is possible to interactively explore the effects of parameter value changes using all previously mentioned visualizations and further statistical values provided by the system. For instance, for unknown data sets it is possible to verify if parameter choices for microsaccades exploration were appropriate. Additionally, we showed that our system can handle both monocular and binocular eye movement analysis and it is not limited to one type only. It is up to the researcher to handle noise in pre-processing and to verify if detected microsaccades are correct. Aligned with our idea to support reproducibility, critical readers of research reports who want to verify the plausibility of suggested research results benefit from such a system. While there is an increase in sharing raw data when publishing research, it additionally requires a software system to reanalyze the data or to reproduce the results. With VisME, researchers will be able to check for themselves if applied parameter settings or filters are appropriate or if there might be other settings that would better fit the data.

### Usability Study

We performed a usability study with a think aloud protocol analysis (21) to collect qualitative feedback on the usefulness and usability of VisME for eye movement researchers. First, participants completed a questionnaire on their research background. Next, we introduced them to our system by explaining VisME’s main features. Subsequently, participants used the system to explore the painting data set of our case study. We provided a list of tasks to guide the participants’ use of the system and to support their awareness of available features. Nonetheless, participants were also encouraged to freely explore the use of the system for whichever aspects interested them. The participants were able to explore how adjusting different parameter values influenced derived microsaccadic statistics by observing how doing so impacted the different visualizations. Some participants asked for specific features and if they were available in the system; we demonstrated them if such features were already implemented. Finally, the participants were asked to complete a standardized questionnaire on usability (10) that was extended to include some eye tracking specific questions. Each question was rated on a Likert scale from 1 (strongly disagree) to 5 (strongly agree). Participants could provide additional text feedback and suggestions for future improvements.

We conducted the user study with 12 voluntary and independent eye tracking experts (3 women and 9 men; 11 participants were between 20 and 39 years old and one was at least 50 years old) who are not authors of this paper. The background of our participants includes computer science, physics, and neuroscience. We wanted to have eye tracking experts with different goals in their research and especially some who are working with microsaccades. The eye tracking researchers belong to four different research groups with specializations on different eye tracking and eye movement related areas. These areas include the analysis of microsaccades, the relationship of eye movements and neuroscience, the development of medical devices to enhance vision, and the development of new eye movement related algorithms. Three of our participants had more than five years of experience with eye tracking, four between three and five years, and five less than three years. Eight of the researchers rated their eye tracking experience from 4 (high) to 5 (very high) on a scale from 1 to 5 (mean: 3.9). Proficiency with microsaccades varied among participants: Four stated to have high and very high experience (4 and 5), whereas six stated to have little and very little experience (1 and 2); the mean value was 3. More than half of all participants claimed to use visualizations often in their analysis. 

Before starting the actual experiment, we asked the participants about their understanding of microsaccades and how they would filter for them. Depending on their proficiency with microsaccades, they could provide detailed information. Most researchers mentioned that microsaccades are located within fixations. The maximum amplitude of microsaccades was stated ranging from 0.5° up to 2° by different participants but most of them employ a threshold of 1°. Furthermore, some mentioned that a manual inspection of each detected microsaccade is very important. For the detection of microsaccades, the algorithm by Engbert and Kliegl (2003b) was named by a few researchers, which confirms that our initial algorithm choice was appropriate. For some researchers, it is very important that the detection is done on binocular data while others use monocular data. This also shows that it is important to support both types of data analysis in our system. Additionally, we asked participants how they would process eye tracking data to explore microsaccade distributions. Most of them described similar approaches to the one we realized in VisME. Additionally, they would verify the data quality at the beginning. Most participants would use MATLAB as analysis software.

All participants liked our system, were able to use it without any problems, and gave a lot of positive feedback. They agreed that our system could be beneficial for teaching purposes (mean: 4.9) and that the eye tracking community would benefit from such a tool (mean: 4.3). The question if participants would prefer to use this software over the steps they described initially for exploring microsaccades had a mean value of 3 and the highest standard deviation. This roughly correlates with the proficiency with microsaccades analysis. Researchers who have less experience were more likely to choose this software. All researchers agreed that VisME served its intended purpose of rendering the analysis of microsaccades more reliable and transparent. 

In particular, the interactive and visual features of our system were received positively. The possibility to click on fixations that are then highlighted in both time and space, and the possibility to scroll through trials were highlighted as a preferred feature. Many researchers stated that VisME is especially useful to get a quick overview of the data.

Recommendations for additional features varied widely depending on the participants’ research background and interests. For a system supporting the whole analysis process, participants suggested that our system should be able to deal with pre-processing steps like data smoothing or processing of eye blinks as well; currently, pre-processing of raw data has to be performed externally. Furthermore, manual inspection of microsaccades is very important. Therefore, manual correction of both fixation and microsaccade areas would be required to adapt detected eye movements that were not marked correctly. Additionally, analysis related to specific events is very important, especially in controlled experiments. In our system, it is possible to visualize positions of events, but it is not possible to consider temporal aspects related to the events in the analysis. Currently, it is possible to analyze microsaccade directions in relation to neighboring fixations; two participants asked for an extension to allow this analysis to be performed in relation to arbitrary target positions on the stimulus (e.g., the center of an image). As there are many different approaches available to detect microsaccades, some participants wished support of further algorithms.

## Discussion

We present a visual analytics system for exploring eye tracking data, with focus on microsaccade analysis. With this system, eye movement researchers will be able to explore and understand microsaccade distributions in time and space. In particular, the interactive nature of the visualizations, namely the ability to vary multiple parameter values allows researchers to determine how sensitive their findings are to parameter variations. This system is tailored for research purposes in that it allows researchers to analyze microsaccadic patterns on the level of participants, trials, and test conditions. It also allows for flexible adjustments of parametric values across these levels in order to account for huge individual differences, if necessary.

Our system will allow for more transparent discourse between researchers and increase the value of public data sets; it will support reproducibility and promote open research. Eye movement researchers will be able to decide for themselves if appropriate parameter values were used as well as to discover unexpected eye movement behavior or verify novel hypotheses on old data. Our system is especially helpful to get an overview of available data sets and provides a simple approach to explore microsaccades for researchers with little experience. In addition, it can serve as an instruction system to help researchers better understand microsaccade movements and issues in their detection. While there remain many possible additions for the application, we believe that eye movement researchers will profit from its visual analytics features to explore microsaccades.

Based on the informal recommendations of eye movement researchers and the feedback from our user study, we have identified some aspects of the current implementation that can be improved as well as features that might be appreciated in future versions; many suggestions have already been mentioned in the results of the user study.

To begin, we implemented the algorithm by Engbert and Kliegl (2003b) for both microsaccade and saccade detection. However, others might be interested in employing different filters for microsaccades (e.g., 49; 45; 5). The introduction of plug-ins would allow researchers to more easily introduce alternative filters. Additionally, auxiliary algorithms exist explicitly to identify eye movements such as square wave jerks. Such algorithms can be easily introduced to the current implementation as an additional and optional processing step.

Currently, the direction vectors between consecutive fixations are defined in terms of their centroids. It has been suggested that we could allow for this direction vector to be flexibly defined – for example, in terms of the last data point of a fixation and the first data point of the subsequent fixation.

The current microsaccades filter assumes that a participant has the same distance to the display for all trials; this distance is used to compute visual angles required for detecting microsaccades. An alternative that would be more accurate is the use of 3D eye position data of each time step (which is provided by, e.g., the Tobii Pro Spectrum) to determine more precise visual angle values for every time step that then can be used to get more reliable results with the eye movement filters.

Furthermore, we intend to improve the visualization of microsaccadic distributions by introducing temporal aspects. This will allow us to visualize variations between early, mid, and late microsaccades. Speculatively, this distinction could result in different patterns – for example, late microsaccades might be more predictive of the following fixation.

### Ethics and Conflict of Interest

The author(s) declare(s) that the contents of the article are in agreement with the ethics described in http://biblio.unibe.ch/portale/elibrary/BOP/jemr/ethics.html and that there is no conflict of interest regarding the publication of this paper. 

### Acknowledgements

This work was funded by the Deutsche Forschungsgemeinschaft (DFG, German Research Foundation) – Project-ID 251654672 – TRR 161 (Projects B01 and C06) and under Germany’s Excellence Strategy – EXE-2075 – 390740016. The authors wish to thank Jeremy Wolfe, Harvard Medical School, and Michelle R. Greene, Bates College, for their permission to use their data set to exemplify the application. Furthermore, the authors would like to thank all researchers who participated in their user study or gave informal feedback to their system.
